# Risk factors associated with deep vein thrombosis in COVID‐19 patients

**DOI:** 10.1002/mco2.52

**Published:** 2021-03-18

**Authors:** Bin Wang, Li Zhang, Shanye Yin, Wenjun Deng, Mingxing Xie

**Affiliations:** ^1^ Department of Ultrasound Union Hospital Tongji Medical College Huazhong University of Science and Technology Wuhan China; ^2^ Hubei Province Key Laboratory of Molecular Imaging Wuhan China; ^3^ Department of Medical Oncology Dana Farber Cancer Institute, Harvard Medical School Boston Massachusetts USA; ^4^ Department of Neurology Massachusetts General Hospital Harvard Medical School Boston Massachusetts USA

## Graphical abstract

Here we identified age, D‐Dimer and CK‐MB as independent risk factors of DVT in COVID‐19 patients. The combination of age, D‐Dimer and CK‐MB yielded a sensitivity of 79.81% and a specificity of 73.20% to predict DVT events.

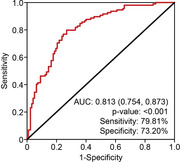

Dear Editor,

The ongoing coronavirus disease 2019 (COVID‐19) pandemic has become the greatest threat to the public health of this generation. Patients infected with severe acute respiratory syndrome coronavirus 2 have a high chance of developing acute respiratory distress syndrome (ARDS) and the mortality rate is high.^1^, ^2^ Risk factors associated with mortality include age, neutrophilia, organ abnormality, and coagulation dysfunction (e.g., elevated D‐dimer).^1^ Consistent with previous findings that viral infections are able to induce thrombosis in human and a broad range of animal hosts,^3^ elevated coagulation function in COVID‐19 patients might enhance the risk of venous thromboembolic events, leading to multifactorial thrombotic disorder such as deep venous thrombosis (DVT) and pulmonary embolism (PE), occasionally as thrombosis of the hepatic, portal, or splanchnic veins. However, the risk factors associated with thrombus formation remained largely indeterminate. Understanding the underlying causes of thrombosis can have important implications for choice and dosing of antithrombotic interventions to enhance diagnostic care and patient management.

In this single‐center study, we retrospectively analyzed the bedside ultrasound findings in COVID‐19 patients with advanced disease admitted to Union Hospital of Tongji Medical College (Wuhan, China) from January 29 to April 11, 2020. Severe cases were defined as tachypnea (≥30 breaths/min), oxygen saturation at rest ≤93%, or PaO2/FIO2 < 300 mm Hg; critical cases (ICU) were defined as respiratory failure requiring mechanical ventilation, shock, or nonrespiratory organ failure. Bedside ultrasound examine was performed upon order by the physicians. An ultrasound scanner equipped with an L12‐5/S5‐1 probe (EPIQ 7C, Philips Medical Systems, Andover, MA, USA,) or a Mindray portable Ultrasound (M9, GD, China, equipped with an L10‐3 probe) had been used to examine bilateral common femoral, deep, and superficial femoral, and the popliteal veins as well as the posterior tibial, peroneal, and calf muscle veins. Clinic features, laboratory findings, and outcomes of the patients were collected during hospitalization and analyzed by two independent analysts.

Altogether 235 COVID‐19 patients were recruited in this study, including 131 (55.7%) men and 104 (44.3%) women, and the mean age was 67.0 years (SD 11.3). Of them, 154 (65.5%) patients were in severe/critical condition and 71 (30.2%) were dead. The mean time from symptom onset to hospital admission was 12.7 ± 7.8 days. The major onset symptoms included fever (193 [82.1%]), dry cough (154 [65.5%]), dyspnea (123 [52.3%]), ARDS (114 [48.5%]), respiratory failure (71 [30.2%]), chest tightness and chest pain (79 [33.6%]), fatigue (139 [59.1%]), headache (18 [7.7%]), and diarrhea (29 [12.3%]).

Bedside ultrasound has been used to performed lower limb vascular examination for 204 patients, and DVT had been observed in 104 (50.1%) of them. As shown in Table S1, we observed no significant difference in gender, body mass index (BMI), or days from symptom onset to hospital admission between the DVT group and non‐DVT group. In contrast, patients with DVT were associated with older ages (67.0 ± 11.3 vs. 59.7 ± 15.5, *p *< 0.001) and longer bedridden time (> 72 h, 69 [66.3%] vs. 36 [36.0%], *p *< 0.001) compared to non‐DVT patients. Patients with DVT had a worse prognosis than patients without DVT, that is, higher chances to develop ARDS (82 [78.8%] vs. 46 [46.0%], *p *= 0.025), severe illness (56 [53.8%] vs. 38 [38.0%], *p *< 0.001), and death (38 [36.5%] vs. 12 [12.0%], *p *< 0.001). Laboratory test suggested that compared to the non‐DVT group, the DVT group had reduced number of palates (204.2 ± 99.8 vs. 33.5 ± 102.1, × 10^9^/L, *p *= 0.040), elevated neutrophil (8.4 ± 5.6 vs. 5.8 ± 3.7, × 10^9^/L, *p *= 0.040), as well as reduced number of lymphocytes (<1.1 × 10^9^/L) (75 [72.1%] vs. 56 [56.0%], *p *= 0.025). Coagulation test screening showed that the DVT group had higher levels of D‐dimer (4.8 ± 3.5 vs. 2 ± 2.5 μg/ml, *p *< 0.001) and prolonged prothrombin time (>16 s) (26 [25.0%] vs. 13 [13.0%], *p *= 0.041). Moreover, patients with DVT showed higher levels of cardiac injury markers, that is, creatine kinase myocardial band (CK‐MB, > 25 U/L) (32 [30.8%] vs. 11 [11.0%], *p *< 0.001) and cardiac troponin I (>26.5 ng/ml) (55 [52.9%] vs. 28 [28.0%], *p *< 0.001). Anticoagulation decision was then made by the treating physician. Patients with DVT were more likely to be treated with glucocorticoid therapy (64 [61.5%] vs. 39 [39.0%], *p *= 0.002) and low molecular weight heparin, both before (*p = *0.002) and after ultrasound diagnosis (*p *< 0.001).

We then used univariate and multivariate logistic regression models to identify the risk factors associated with DVT. As shown in Table 1, univariate analysis revealed that risk factors associated with DVT included age, disease severity, death, bedridden time, diastolic blood pressure (DBP), the numbers of platelet, white blood cells, neutrophil, and lymphocytes, as well as the levels of D‐dimer, RT, activated partial thromboplastin time, CK‐MB, and cardiac troponin I level. In the multivariant analysis model, older age (odds ratio [OR] [95% confidence interval (CI)], 1.04 [1.011, 1.064]; *p *= 0.005), high levels of D‐dimer at admission (>1.0 μg/ml) (OR [95% CI], 6.08 [2.444, 15.10]; *p *< 0.001), and elevated CK‐MB (> 25 U/L) (OR [95% CI], 2.55 [1.036, 6.260]; *p *= 0.042) were independent risk factors associated with DVT. Using a receiver‐operating characteristic analysis, a combination of age, D‐dimer, and CK‐MB yielded a sensitivity of 79.81% and a specificity of 73.20% for prediction of DVT (Figure S1).

**TABLE 1:** Univariate and multivariate logistic regression models to identify the risk factors associated with DVT

Variable	Univariable OR (95% CI)	*p*‐Value	Multivariable OR (95% CI)	*p*‐Value
Age (years)	1.041 (1.019, 1.064)	<0.001	1.038 (1.011, 1.064)	0.005
Male sex	1.536 (0.883, 2.674)	0.13		
BMI (kg/m^2^)	0.994 (0.890, 1.110)	0.92		
Disease severity status				
General	1.000 (ref)			
Severs/critical	4.375 (2.370, 8.079)	<0.001		
Death	4.222 (2.049, 8.702)	<0.001		
Symptom onset to hospital admission (days)	0.974 (0.939, 1.010)	0.15		
Bedridden time				
≤72 h	1.000 (ref)			
>72 h	3.505 (1.970, 6.237)	<0.001		
Respiratory rate (breaths per min)	1.015 (0.970, 1.062)	0.52		
Temperature (°C)	1.150 (0.901, 1.467)	0.26		
Heart rate (beats per min)	1.005 (0.989, 1.021)	0.54		
SBP (mm Hg)	0.994 (0.980, 1.009)	0.45		
DBP (mm Hg)	0.974 (0.952, 0.997)	0.026		
BSA	0.848 (0.197, 3.653)	0.82		
Hemoglobin (g/L)	0.992 (0.979, 1.006)	0.26		
Platelets (×10^9^/L)				
125‐350	1.000 (ref)			
<125	2.557 (1.101, 5.940)	0.029		
>350	0.996 (0.400, 2.483)	0.99		
White blood cells (×10^9^/L)				
3.5‐9.5	1.000 (ref)		1.000 (ref)	
<3.5	0.685 (0.121, 3.877)	0.67	1.702 (0.140, 20.69)	0.68
>9.5	2.990 (1.617, 5.528)	<0.001	0.975 (0.348, 2.733)	0.96
Neutrophil (×10^9^/L)				
1.8‐6.3	1.000 (ref)		1.000 (ref)	
<1.8	0.653 (0.121, 3.533)	0.62	0.364 (0.024, 5.452)	0.46
>6.3	3.164 (1.767, 5.667)	<0.001	1.843 (0.688, 4.939)	0.22
Lymphocytes (×10^9^/L)				
1.1‐3.2	1.000 (ref)			
<1.1	2.057 (1.142, 3.705)	0.016		
>3.2	1.536 (0.092, 25.57)	0.76		
D‐Dimer (μg/ml)				
<0.5	1.000 (ref)		1.000 (ref)	
0.5‐1.0	1.133 (0.390, 3.292)	0.82	1.152 (0.355, 3.739)	0.81
>1.0	7.310 (3.289, 16.25)	<0.001	6.075 (2.444, 15.10)	<0.001
Prothrombin time (s)				
11‐16	1 (ref)			
<11	0 (0, 0)	1.00		
>16	2.179 (1.047, 4.537)	0.037		
Activated partial thromboplastin time (s)				
27‐45	1.000 (ref)			
<27	2.290 (0.203, 25.81)	0.50		
>45	1.989 (1.038, 3.810)	0.038		
Creatine kinase myocardial band (U/L)				
≤25	1.000 (ref)		1.000 (ref)	
>25	3.556 (1.675, 7.546)	<0.001	2.546 (1.036, 6.260)	0.042
Cardiac troponin I (ng/ml)				
≤26.5	1.000 (ref)		1.000 (ref)	
>26.5	2.886 (1.613, 5.166)	<0.001	1.602 (0.792, 3.242)	0.19
B‐type natriuretic peptide (pg/ml)				
≤100	1.000 (ref)			
>100	1.474 (0.804, 2.701)	0.21		

*Note*: Significant risk factors from univariate analysis are put in multivariate analysis. SBP, systolic blood pressure, DBP, diastolic blood pressure, BSA, bovine serum albumin.

Together, our results revealed that the viral infection was associated with a transient increased risk of venous thromboembolic events. DVT had been observed in half of the patients, particularly for those who were critically ill, in line with previous studies showing an overall rate of 69% in a French cohort.^4^ The actual DVT rate could be even higher as the bedside ultrasound examines had only been performed for a few times, highlighting the important role of DVT in COVID‐19 pathogenesis. DVT might lead to more severe thromboembolic diseases such as PE, resulting in increased mortality. Moreover, patients with a history of acute ischemic stroke (AIS) and/or its risk factors are particularly at‐risk. In line with this notion, AIS had been observed in 5.7% of the severe cases, which can be immediately linked to increased mortality and poor outcomes, indicating the need of active anticoagulant therapy. Although coagulation test screening (i.e., measurement of D‐dimer and fibrinogen levels) is indicative of increased risk of DVT, current guideline do not suggest the use of full‐intensity anticoagulation doses unless otherwise clinically indicated. In addition to DVT, myocardial injury is a common phenomenon in patients with COVID‐19 as indicated by elevated levels of CK‐MB. Our findings that elder age, high levels of D‐dimer, and CK‐MB are independently associated with DVT, particularly in individuals with preexisting cardiovascular diseases, providing implications for choice, dosing, and laboratory monitoring of antithrombotic therapies. Our results suggested that early intervention and thromboembolic prophylaxis should be considered for those patients.

## CONFLICT OF INTEREST

The authors declare that there is no conflict of interest.

## AUTHOR CONTRIBUTIONS

Bin Wang and Li Zhang collected the clinical data. Shanye Yin and Wenjun Deng processed statistical data. Shanye Yin and Wenjun Deng drafted and revised the manuscript. Mingxing Xie designed and guided the study.

## ETHICS STATEMENT

The study was approved by Union hospital Tongji Medical College, Huazhong University of Science and Technology Ethics Committee (KY‐2020‐02.06) and written informed consent was waived for emerging infectious diseases.

### DATA AVAILABILITY STATEMENT

The authors confirm that the data supporting the findings of this study are available within the supplementary materials and are available on request from the corresponding author.

## Supporting information

Supporting information
