# Supplementary material for: Risk factors associated with deep vein thrombosis in COVID‐19 patients
Source: MedComm (2020). 2021 Mar 18;2(2):288–91. doi: 10.1002/mco2.52 (PMC8251315; doi:10.1002/mco2.52)

Table S1. Clinical Characteristics and laboratory data of DVT *vs*. non-DVT Patients with COVID-19

**Clinical feature**	**Total (n=235)**	**Non-DVT (n=100)**	**DVT (n=104)**	**P-value**
Age (years)	64.1 ± 13.7	59.7 ± 15.5	67.0 ± 11.3	<0.001
≤ 44, n (%)	24 (10.2)	18 (18.0)	5 (4.8)	<0.001
45-64, n (%)	80 (34.0)	40 (40.0)	30 (28.8)	
≥ 65, n (%)	131 (55.7)	42 (42.0)	69 (66.3)	
Male sex, n (%)	131 (55.7)	49 (49.0)	62 (59.6)	0.16
BMI (kg/m2)	23.7 ± 2.9	23.8 ± 2.9	23.7 ± 2.8	0.92
< 18.5, n (%)	6 (3.6)	4 (5.1)	0 (0)	0.24
18.5-24.9, n (%)	108 (63.9)	46 (59.0)	52 (68.4)	
25-29.9, n (%)	50 (29.6)	26 (33.3)	22 (28.9)	
≥ 30, n (%)	5 (3.0)	2 (2.6)	2 (2.6)	
Exposure History, n (%)	12 (5.1)	5 (5.0)	4 (3.8)	0.74
Disease severity status				<0.001
General, n (%)	81 (34.5)	54 (54.0)	22 (21.2)	
Severe/Critical, n (%)	154 (65.5)	46 (46.0)	82 (78.8)	
Hospital stay (Days)	28.7 ± 15.0	27.7 ± 13.8	31.5 ± 15.9	0.065
Death, n (%)	71 (30.2)	12 (12.0)	38 (36.5)	<0.001
Symptom onset to hospital admission (days)	12.7 ± 7.8	13.5 ± 9.1	11.9 ± 6.4	0.15
Bedridden time				<0.001
≤ 72 hours, n (%)	108 (46.0)	64 (64.0)	35 (33.7)	
> 72 hours, n (%)	127 (54.0)	36 (36.0)	69 (66.3)	
Onsite symptoms				
Fever, n (%)	193 (82.1)	84 (84.0)	84 (80.8)	0.59
Dry cough, n (%)	154 (65.5)	69 (69.0)	67 (64.4)	0.55
Dyspnea, n (%)	123 (52.3)	51 (51.0)	60 (57.7)	0.40
ARDS, n (%)	114 (48.5)	38 (38.0)	56 (53.8)	0.025
Respiratory failure, n (%)	71 (30.2)	21 (21.0)	34 (32.7)	0.082
Chest tightness and chest pain, n (%)	79 (33.6)	36 (36.0)	38 (36.5)	1.00
Fatigue, n (%)	139 (59.1)	58 (58.0)	67 (64.4)	0.39
Headache, n (%)	18 (7.7)	11 (11.0)	4 (3.8)	0.062
Diarrhea, n (%)	29 (12.3)	10 (10.0)	16 (15.4)	0.30
Shock, n (%)	5 (2.1)	1 (1.0)	2 (1.9)	1.00
Acute gastrointestinal bleeding, n (%)	7 (3.0)	1 (1.0)	2 (1.9)	1.00
Vital signs				
Respiratory rate (breaths per min)	24.7 ± 6.1	24.5 ± 5.8	25.0 ± 6.3	0.52
Temperature (℃)	38.2 ± 1.1	38.2 ± 1.1	38.3 ± 1.1	0.26
Heart rate (beats per min)	91.1 ± 17.8	90.8 ± 17.0	92.3 ± 18.2	0.54
SBP (mmHg)	132.6 ± 19.6	133.5 ± 19.5	131.6 ± 17.6	0.46

DBP (mmHg)	80.2 ± 12.7	82.6 ± 12.5	78.6 ± 12.4	0.024
Laboratory				
BSA	1.6 ± 0.3	1.7 ± 0.2	1.7 ± 0.2	0.83
Hemoglobin (g/L)	116.2 ± 22.4	119.9 ± 20.8	116.6 ± 20.9	0.26
Platelets ($\times 10^9$/L)	214.0 ± 102.0	233.5 ± 102.1	204.2 ± 99.8	0.040
< 125, n (%)	40 (17.0)	9 (9.0)	21 (20.2)	0.079
125-350, n (%)	172 (73.2)	80 (80.0)	73 (70.2)	
> 350, n (%)	23 (9.8)	11 (11.0)	10 (9.6)	
White blood cells ($\times 10^9$/L)	11.0 ± 27.0	8.3 ± 9.3	14 ± 39.4	0.16
< 3.5, n (%)	8 (3.4)	4 (4.0)	2 (1.9)	<0.001
3.5-9.5, n (%)	141 (60.0)	74 (74.0)	54 (51.9)	
> 9.5, n (%)	86 (36.6)	22 (22.0)	48 (46.2)	
Neutrophil ($\times 10^9$/L)	7.3 ± 4.8	5.8 ± 3.7	8.4 ± 5.6	<0.001
< 1.8, n (%)	8 (3.4)	5 (5.0)	2 (1.9)	<0.001
1.8-6.3, n (%)	108 (46.0)	62 (62.0)	38 (36.5)	
> 6.3, n (%)	119 (50.6)	33 (33.0)	64 (61.5)	
Lymphocytes ($\times 10^9$/L)	2.6 ± 21.9	1.8 ± 7.3	4 ± 32.1	0.52
< 1.1, n (%)	155 (66.0)	56 (56.0)	75 (72.1)	0.025
1.1-3.2, n (%)	78 (33.2)	43 (43.0)	28 (26.9)	
> 3.2, n (%)	2 (0.9)	1 (1.0)	1 (1.0)	
D-Dimer (μg/ml)	3.5 ± 3.3	2 ± 2.5	4.8 ± 3.5	<0.001
< 0.5, n (%)	48 (20.6)	34 (34.7)	10 (9.6)	<0.001
0.5-1.0, n (%)	34 (14.6)	24 (24.5)	8 (7.7)	
> 1.0, n (%)	151 (64.8)	40 (40.8)	86 (82.7)	
Prothrombin time (sec)	15.3 ± 5.0	14.5 ± 4.7	15.4 ± 3.9	0.11
< 11, n (%)	1 (0.4)	1 (1.0)	0 (0)	0.041
11-16, n (%)	183 (78.2)	85 (85.9)	78 (75.0)	
> 16, n (%)	50 (21.4)	13 (13.0)	26 (25.0)	
Activated partial thromboplastin time (sec)	41.5 ± 13.2	39.1 ± 11.4	42.1 ± 13.2	0.077
< 27, n (%)	5 (2.1)	1 (1.0)	2 (1.9)	0.070
27-45, n (%)	162 (69.2)	79 (79.8)	69 (66.3)	
> 45, n (%)	67 (28.6)	19 (19.2)	33 (31.7)	
Coagulation dysfunction, n (%)	59 (25.1)	16 (16.0)	29 (27.9)	0.044
Creatine Kinase Myocardial Band (U/L)	23.8 ± 55.1	13.0 ± 20.6	24.8 ± 48.7	0.027
≤ 25, n (%)	178 (76.1)	88 (88.9)	72 (69.2)	<0.001
> 25, n (%)	56 (23.9)	11 (11.0)	32 (30.8)	
Cardiac troponin I (ng/mL)	380.6 ± 1578.3	219.7 ± 1226.9	340.3 ± 1605.5	0.58
≤ 26.5, n (%)	130 (55.3)	72 (72.0)	49 (47.1)	<0.001
> 26.5, n (%)	105 (44.7)	28 (28.0)	55 (52.9)	
B-type natriuretic peptide (pg/mL)	243.0 ± 391.6	211.3 ± 362.8	230.7 ± 424.3	0.76
≤ 100, n (%)	113 (55.7)	55 (64.7)	51 (55.4)	0.22
> 100, n (%)	90 (44.3)	30 (35.3)	41 (44.6)	

Treatment				
Antiviral therapy, n (%)	223 (95.3)	95 (95.0)	101 (97.1)	0.49
Antibiotic therapy, n (%)	194 (83.3)	77 (77.8)	91 (87.5)	0.094
Glucocorticoid therapy, n (%)	122 (52.1)	39 (39.0)	64 (61.5)	0.002
Immunoglobulin, n (%)	93 (40.1)	28 (28.6)	51 (49.0)	0.004
Low-molecular-weight heparin, n (%)	141 (60.3)	34 (34.0)	86 (82.7)	<0.001
LMWH before ultrasound for DVT, n (%)	79 (42.5)	24 (28.9)	46 (52.9)	0.002
LMWH after ultrasound for DVT, n (%)	85 (45.7)	22 (26.5)	59 (67.8)	<0.001
Aspirin, n (%)	23 (9.8)	8 (8.0)	11 (10.6)	0.63
Aspirin before ultrasound for DVT, n (%)	14 (7.4)	5 (6.0)	7 (8.0)	0.77
Aspirin after ultrasound for DVT, n (%)	11 (5.9)	4 (4.8)	5 (5.8)	1.00

Figure S1. Receiver-operating characteristic (ROC) analysis of the sensitivity and a specificity of a combination of age, D-Dimer and CK-MB for prediction of DVT.

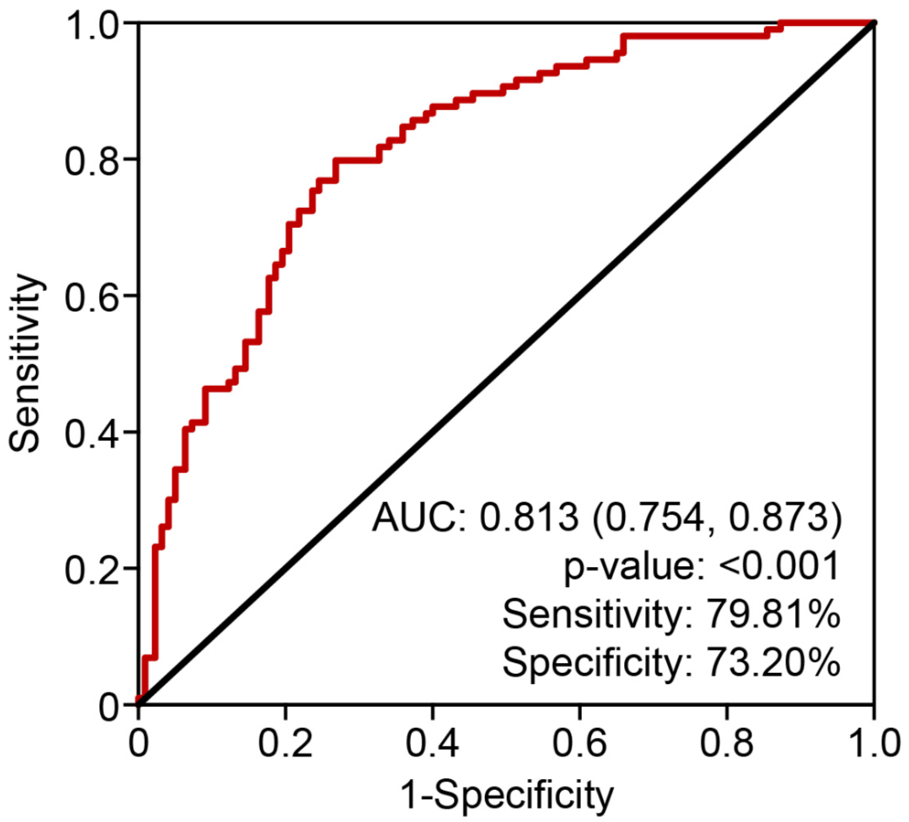

Supplement: Supplementary file 1 — Supporting information [file MCO2-2-288-s001.pdf]
